# HMGB1 Post-Translational Modifications in Epstein–Barr Virus-Associated Nasopharyngeal Carcinoma: Current Evidence, Emerging Mechanistic Concepts, and Unresolved Questions

**DOI:** 10.3390/ijms27188196

**Published:** 2026-09-15

**Authors:** Cho Yiu Lau, Khadija Shahed Khan, Dora Lai-Wan Kwong, Wei Dai, Ngar Woon Kam

**Affiliations:** 1 School of Pharmacy, Faculty of Medicine, The Chinese University of Hong Kong, Sha Tin, Hong Kong, China; yoyo22@link.cuhk.edu.hk; 2 School of Biomedical Sciences, Faculty of Medicine, The Chinese University of Hong Kong, Sha Tin, Hong Kong, China; 1155165368@link.cuhk.edu.hk; 3 Centre of Cancer Medicine, Department of Clinical Oncology, School of Clinical Medicine, LKS Faculty of Medicine, The University of Hong Kong, Hong Kong, China; dlwkwong@hku.hk

**Keywords:** HMGB1, nasopharyngeal carcinoma, post-translational modifications, immune regulation, tumor microenvironment, EBV, therapeutics

## Abstract

Nasopharyngeal carcinoma (NPC) is a geographically distinct malignancy closely associated with Epstein–Barr virus (EBV) infection and characterized by extensive epigenetic reprogramming within a highly immunosuppressive tumor microenvironment (TME). High-mobility group box 1 (HMGB1), a multifunctional chromatin-binding protein that can also act as an extracellular damage-associated molecular pattern (DAMP), has emerged as an important regulator of genome organization, transcriptional control, cellular stress responses, and immune signaling. Increased HMGB1 expression has been reported in NPC and is associated with adverse clinicopathological features and poor patient outcomes. Emerging evidence suggests that the diverse biological functions of HMGB1 are influenced by post-translational modifications (PTMs), which affect its subcellular localization, molecular interactions, and extracellular signaling functions. Through these regulatory mechanisms, HMGB1 may transition from a nuclear chromatin-associated protein to an extracellular mediator of immune and inflammatory responses. PTMs including acetylation, phosphorylation, glycosylation, oxidation, methylation, and lactylation have been implicated in regulating HMGB1 trafficking and function, although the specific roles of many of these modifications in NPC remain incompletely characterized. In this review, we summarize current evidence regarding HMGB1 PTMs and discuss their potential implications for EBV-associated NPC, with emphasis on nuclear regulation, immune crosstalk, and therapeutic response. We explicitly distinguish findings directly demonstrated in NPC from mechanistic insights derived from other malignancies and related disease models, and identify areas where proposed mechanisms remain hypothesis-generating rather than experimentally validated in NPC. By integrating current evidence with emerging mechanistic concepts, we highlight key knowledge gaps, unresolved questions, and priorities for future research. A better understanding of PTM-dependent HMGB1 regulation may facilitate the development of novel biomarker and therapeutic strategies for EBV-associated NPC.

## 1. Introduction

Nasopharyngeal carcinoma (NPC) is a distinct epithelial malignancy arising from the mucosal lining of the nasopharynx. According to the current WHO classification, NPC is categorized as keratinizing squamous cell carcinoma, non-keratinizing carcinoma (including differentiated and undifferentiated subtypes), and the rare basaloid squamous cell carcinoma. Historically, these entities correspond approximately to WHO type I (keratinizing), type II (non-keratinizing differentiated), and type III (non-keratinizing undifferentiated) NPC. Non-keratinizing NPC (primarily the differentiated and undifferentiated subtypes) comprises over 95% of cases in endemic areas such as Southern China and Southeast Asia [1]. Unlike other head and neck cancers that are predominantly associated with alcohol and tobacco consumption, almost all cases of type II and III NPC are etiologically associated with Epstein–Barr virus (EBV) infection.

Within the host, EBV can establish two alternative life cycle modes, latent or lytic, where the switch from latency to the active lytic cycle is termed EBV reactivation [2]. Following latent EBV infection and the subsequent clonal expansion of infected epithelial cells, the virus drives tumor survival and proliferation by coordinating the expression of viral oncogenes, including Epstein–Barr nuclear antigen 1 (EBNA1), latent membrane proteins 1 and 2 (LMP1/2), and various viral non-coding RNAs. These viral factors also drive genomic instability by promoting aberrant host epigenetic alterations, including DNA methylation and histone modifications; for instance, EBNA1 and LMP1 directly interact with host DNA methyltransferases (DNMTs) or demethylases to modulate these epigenetic landscapes [3]. In the presence of tumor microenvironment (TME)-associated environmental stressors, such as oxidative stress and severe hypoxia, lytic EBV reactivation is triggered, marked by the rapid upregulation of the immediate-early viral transactivator genes *BZLF1* (encoding the Zta protein) and *BRLF1* (encoding the Rta protein) [2]. This lytic reactivation is associated with a poor clinical prognosis in NPC patients [4]. Furthermore, EBV contributes to immune dysregulation within the TME by recruiting regulatory immune populations and altering chemokine profiles that promote immune evasion [5]. Ultimately, EBV contributes to host epigenetic remodeling and the establishment of a hypoxic, inflammatory TME.

In EBV-infected tumor tissues, high-mobility group box 1 (HMGB1) was reported to be highly expressed in 53.6% of NPC specimens according to the immunohistochemical scoring criteria defined in the original study. Elevated HMGB1 expression was further associated with accelerated NPC cell proliferation [6]. Clinically, upregulated HMGB1 expression is also reported to correlate with advanced malignant status and worse overall survival in NPC patients [7]. HMGB1 exhibits dynamic, compartment-specific functions, including maintaining chromatin architecture in the nucleus, regulating signaling pathways in the cytoplasm, and acting as a damage-associated molecular pattern (DAMP) following extracellular release. These distinct functional states are regulated by multiple post-translational modifications (PTMs), which influence HMGB1 localization, secretion, and signaling in response to metabolic and environmental stresses such as hypoxia, oxidative stress, and altered glycosylation states.

Despite its established association with poor prognosis in NPC, a knowledge gap remains regarding how its subcellular distribution and specific PTM status interact to influence disease pathophysiology and clinical outcomes. Therefore, understanding how PTMs regulate the transition of HMGB1 from a chromatin-associated protein to an extracellular immune mediator may facilitate the identification of novel therapeutic targets and strategies for EBV-associated NPC. Given the limited mechanistic understanding of HMGB1 PTMs in NPC, this review integrates current evidence from NPC and related biological systems to examine how PTM-dependent regulation influences HMGB1 localization, function, and signaling. Particular emphasis is placed on distinguishing mechanisms directly demonstrated in NPC from broader concepts inferred from EBV-associated malignancies, other cancer types, and inflammatory diseases. Through this approach, we highlight emerging mechanistic concepts, knowledge gaps, and priorities for future investigation.

## 2. The Role of HMGB1

In humans, the HMGB1 gene is located on chromosome 13q12 and contains six reported polymorphic loci [8]. HMGB1 is a highly abundant, ~25 kilodalton (kDa) non-histone chromatin protein composed of three structural domains: two tandem DNA-binding domains (HMG Box-A and Box-B) and a highly acidic C-terminal tail. Both the Box-A and Box-B domains are responsible for its non-sequence-specific DNA-binding functions, allowing HMGB1 to intercalate into the minor groove of the DNA double helix. The acidic C-terminal tail regulates DNA-binding affinity through intramolecular interactions with the HMG boxes. While these structural features govern HMGB1 interactions with DNA and protein partners, its dynamic subcellular localization, shuttling between the nucleus, cytosol, and extracellular space, is tightly regulated by PTMs, which will be discussed in detail in Section 3.

### 2.1. Nuclear Functions of HMGB1

Within the nucleus, HMGB1 primarily functions as a chromatin-binding protein and a DNA chaperone. Unlike classical transcription factors that recognize specific consensus DNA sequences, HMGB1 preferentially binds to distorted or structurally altered DNA. It recognizes DNA lesions and selectively interacts with regions exhibiting bent or damaged conformations. By binding to the minor groove of the DNA double helix, HMGB1 further alters local DNA architecture, facilitating DNA bending and promoting damage recognition [9]. HMGB1 has been implicated in multiple DNA damage response and repair pathways, including nucleotide excision repair (NER), mismatch repair (MMR), base excision repair (BER), and DNA double-strand break repair (DSBR) [10]. However, its effects on DNA repair are highly context-dependent and may vary according to the type of DNA lesion, repair pathway, and cellular context. While nuclear HMGB1 contributes to genome maintenance and DNA damage responses in normal cells, aberrant HMGB1 activity in tumor cells may facilitate repair of therapy-induced DNA damage, thereby promoting treatment resistance.

Beyond DNA repair, HMGB1 functions as a key epigenetic regulator. Its ability to induce DNA bending and alter chromatin topology enables modulation of chromatin accessibility and transcriptional activity. HMGB1 facilitates the recruitment and activity of adenosine triphosphate (ATP)-dependent chromatin-remodelling complexes such as SWI/SNF. It can also regulate the epigenome through crosstalk with histone-modifying enzymes, including histone acetyltransferases (e.g., CBP/p300), promoting localized histone acetylation and a transcriptionally permissive chromatin state. In addition, HMGB1 collaborates with linker histone H1 and other regulatory complexes to influence chromatin compaction and gene silencing. Through these mechanisms, HMGB1 translates cellular stress signals into coordinated transcriptional reprogramming, including processes such as epithelial–mesenchymal transition (EMT) and stress-response gene activation.

#### 2.1.1. HMGB1 in EBV-Driven Epigenetic Reprogramming

During EBV-driven oncogenesis, the physiological roles of HMGB1 in maintaining chromatin accessibility and protecting promoter regions from aberrant methylation may become disrupted. EBV infection induces extensive host epigenomic remodeling, leading to a CpG island methylator phenotype (CIMP) characterised by widespread promoter hypermethylation and silencing of tumour suppressor genes. Matsusaka et al. demonstrated that EBV infection in gastric epithelial cell lines induces genome-wide de novo DNA methylation in a time-dependent manner, resulting in targeted silencing of genes such as CXXC4, TIMP2, and PLXND1, thereby contributing to early malignant transformation [11]. These findings suggest that EBV-induced epigenetic remodeling may intersect with HMGB1 biology and influence the transcriptional programs regulated by chromatin accessibility.

This possibility is particularly relevant given the established role of HMGB1 as a chromatin architectural protein. HMGB1 regulates DNA bending, nucleosome dynamics, and chromatin accessibility, thereby facilitating transcription factor binding and gene regulation. Furthermore, HMGB1 preferentially recognizes structurally distorted DNA and participates in the assembly of transcriptional regulatory complexes. Consequently, EBV-induced genome-wide methylation changes may not only alter host gene expression directly but also reshape the chromatin environment in which HMGB1 functions.

Although specific EBV-induced HMGB1 PTMs have not yet been experimentally demonstrated in NPC, available evidence suggests several plausible mechanistic links between EBV-associated signaling and PTM regulation. For example, LMP1-driven metabolic reprogramming may generate a lactate-rich microenvironment that favors HMGB1 lactylation and acetylation, both of which have been associated with altered subcellular localization and nuclear export [12]. Similarly, EBNA1-induced oxidative stress may influence HMGB1 redox states through modification of its redox-sensitive cysteine residues [13]. In addition, EBV-encoded BART microRNAs (miRNAs) may represent another indirect regulatory pathway through their ability to target LMP1 and modulate downstream NF-κB signaling [14]. Whether these viral miRNAs contribute to PTM-dependent regulation of HMGB1 remains unknown and warrants further investigation.

Beyond its role in host chromatin regulation, HMGB1 has also been implicated in the regulation of EBV chromatin states during viral reactivation. Previous studies demonstrated that HMGB1 facilitates the binding of the EBV lytic transactivators ZEBRA and Rta to viral promoters, promoting transcription of lytic genes and supporting viral reactivation [15]. More recently, HMGB1 was shown to sustain BZLF1 (ZEBRA) expression through an NLRP3 inflammasome-dependent pathway. Mechanistically, HMGB1 promotes NLRP3 activation and caspase-1 signaling, resulting in the loss of KAP1-mediated heterochromatin repression at the BZLF1 promoter and maintenance of the lytic transcriptional program [16]. These findings suggest that HMGB1 contributes to both chromatin regulation and epigenetic control of EBV reactivation.

#### 2.1.2. HMGB1 in EBV Reactivation and Oncogenic Signaling

Zhu et al. demonstrated a positive correlation between HMGB1 expression and the viral oncoprotein LMP1 in EBV-positive NPC tissues compared with EBV-negative tissues [6]. However, no EBV-induced HMGB1 PTMs have yet been directly demonstrated in NPC. In addition, expression of the HMGB1 receptor, the receptor for advanced glycation end products (RAGE), was upregulated following EBV infection, and HMGB1 overexpression promoted tumor growth in EBV-infected CNE-2 cells in a RAGE-dependent manner, highlighting the contribution of HMGB1 signaling to EBV-associated tumor progression.

Additional evidence suggests that HMGB1 plays an active role in EBV biology beyond its increased expression. HMGB1 levels are increased in lytically reactivated EBV-positive cells compared with latent cells, and HMGB1 depletion reduces the proportion of ZEBRA-positive cells and impairs maintenance of the lytic program [16]. These findings place HMGB1 functionally upstream of key EBV reactivation pathways and suggest that HMGB1 may contribute to EBV-associated disease progression through sustained viral reactivation. Furthermore, activation of the HMGB1–NLRP3 axis highlights a previously underappreciated connection between danger sensing and EBV pathogenesis. By sustaining NLRP3 inflammasome activity, HMGB1 promotes caspase-1 signaling and supports continued expression of the EBV lytic switch protein ZEBRA [16]. Together, these findings identify HMGB1 as an active regulator of EBV reactivation that links inflammatory signaling with viral gene expression.

Beyond the protein-mediated mechanisms discussed above, EBV-encoded miRNAs may provide an additional layer of HMGB1 regulation. EBV was the first human virus reported to encode viral miRNAs [17,18]. These small non-coding RNAs regulate gene expression through post-transcriptional repression of target mRNAs and play important roles in viral persistence, immune evasion, and oncogenesis [19,20]. Notably, EBV-encoded BART miRNAs are highly expressed in EBV-associated epithelial malignancies and regulate multiple inflammatory and immune signaling pathways [21]. While direct evidence demonstrating regulation of HMGB1 by EBV miRNAs remains limited, several BART miRNAs modulate pathways that overlap with HMGB1-associated signaling networks, including the NLRP3 inflammasome, NF-κB, and cytokine signaling pathways. Consequently, whether EBV-encoded miRNAs influence HMGB1 activity, PTM regulation, or HMGB1-dependent inflammatory responses remains an important area for future investigation.

### 2.2. Cytosolic and Extracellular Functions of HMGB1

In the cytoplasm, HMGB1 mainly functions to regulate autophagy, a catabolic mechanism essential for maintaining cellular health and survival by orchestrating the engulfment and lysosomal degradation of intracellular proteins and organelles [22]. During starvation or metabolic stress, cells utilize this process to reallocate nutrients to vital cellular pathways. Mechanistically, cytosolic HMGB1 binds directly to Beclin-1, prompting the dissociation of the Beclin-1-Bcl-2 complex and inducing autophagy to sustain tissue homeostasis. However, this system is frequently hijacked in malignancies, where upregulated HMGB1-induced autophagy suppresses apoptosis and drives cancer cell survival [23].

Extracellularly, secreted HMGB1 is recognized as a pro-inflammatory alarmin and actively interacts with surrounding immune cell populations to propagate chronic inflammatory signaling [24]. This phenomenon is discussed further in Section 5. Conversely, during acute tissue damage, secreted HMGB1 acts on resident muscle stem cells, hepatocytes, and infiltrating leukocytes to promote muscle and liver regeneration. The functional outcome of extracellular HMGB1 is therefore highly context-dependent and influenced by both local microenvironmental conditions and the molecular state of the protein.

Extracellular HMGB1 may arise through both active secretion from viable cells and passive release following cellular injury or death. These processes are biologically distinct and may generate HMGB1 species with different PTM states, concentrations, binding partners, and immunological consequences [25]. Therefore, HMGB1 released during treatment-induced cell death should not necessarily be considered functionally equivalent to actively secreted HMGB1 derived from stressed inflammatory or tumor cells. Importantly, the biological effects of extracellular HMGB1 are further influenced by its mode of release, molecular state, binding partners, and local microenvironmental context. As a result, distinct extracellular HMGB1 species may elicit divergent immunological and biological responses despite sharing a common origin.

**Figure 1:**
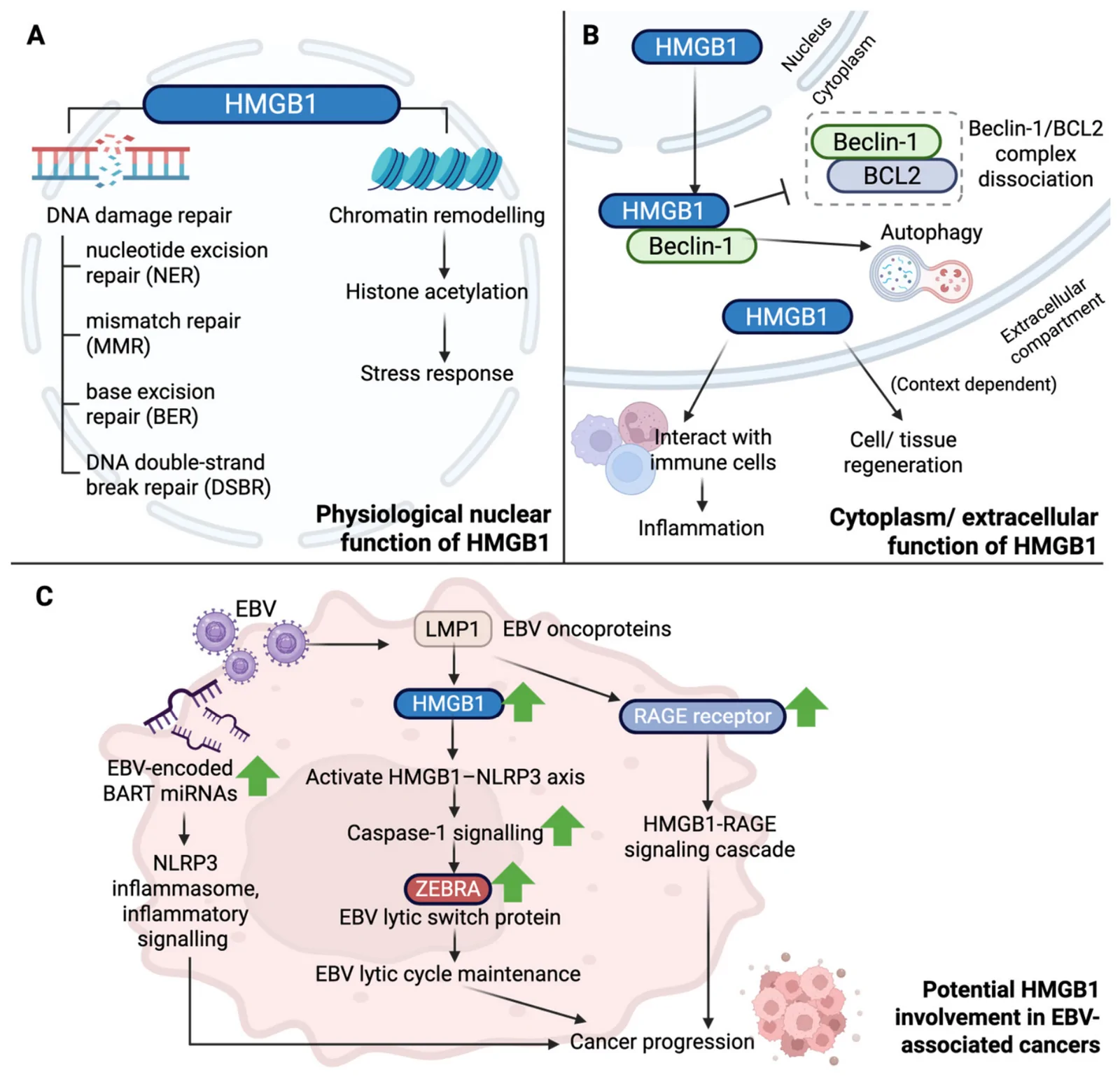
**Physiological functions of HMGB1 and its regulatory involvement in EBV-associated pathogenesis.** (**A**) Within the nucleus, HMGB1 functions as an architectural chromatin-binding protein essential for genomic stability. It coordinates multiple DNA repair pathways and facilitates chromatin remodeling and downstream histone acetylation to orchestrate cellular stress responses. (**B**) Upon translocation to the cytoplasm, HMGB1 binds Beclin-1 following the dissociation of the Beclin-1/BCL2 complex, thereby stimulating autophagic flux. When secreted or passively released into the extracellular compartment, HMGB1 engages immune cells to trigger pro-inflammatory responses or mediates cell and tissue regeneration in a context-dependent manner. (**C**) Epstein–Barr virus (EBV) infection drives the expression of viral oncoproteins (e.g., LMP1), which upregulate both HMGB1 and its cognate receptor, RAGE (green arrows indicate upregulation). Activation of the downstream HMGB1–RAGE signaling cascade directly promotes cancer progression. Concurrently, HMGB1 activates the HMGB1–NLRP3 axis to trigger caspase-1 signaling, sustaining the expression of the EBV lytic switch protein ZEBRA and maintaining the viral lytic cycle. EBV-encoded BART miRNAs further modulate NLRP3 inflammasome activity and inflammatory signaling, intersecting with HMGB1-associated pathways. Green upward arrows indicate increased expression or activation of the indicated molecules/signaling pathways following EBV infection.

It is important to note that HMGB1 function is highly dynamic and influenced by both spatial and temporal contexts, as summarized in Figure 1. Distinct biological activities may arise depending on its subcellular localization (nuclear, cytoplasmic, or extracellular), redox state, and disease stage, including treatment-induced injury, post-radiotherapy inflammation, tumor recurrence, and immunotherapy. Furthermore, HMGB1 signaling is unlikely to be uniform throughout the TME, as hypoxic regions, invasive margins, necrotic areas, and immune-cell-rich niches may contain distinct HMGB1 species and responding cell populations.

To understand how these diverse intracellular and extracellular functions are regulated, it is essential to examine the PTMs that govern HMGB1 localization, secretion, and signaling, as discussed in the following section.

## 3. Post-Translational Modification of HMGB1

**Figure 2:**
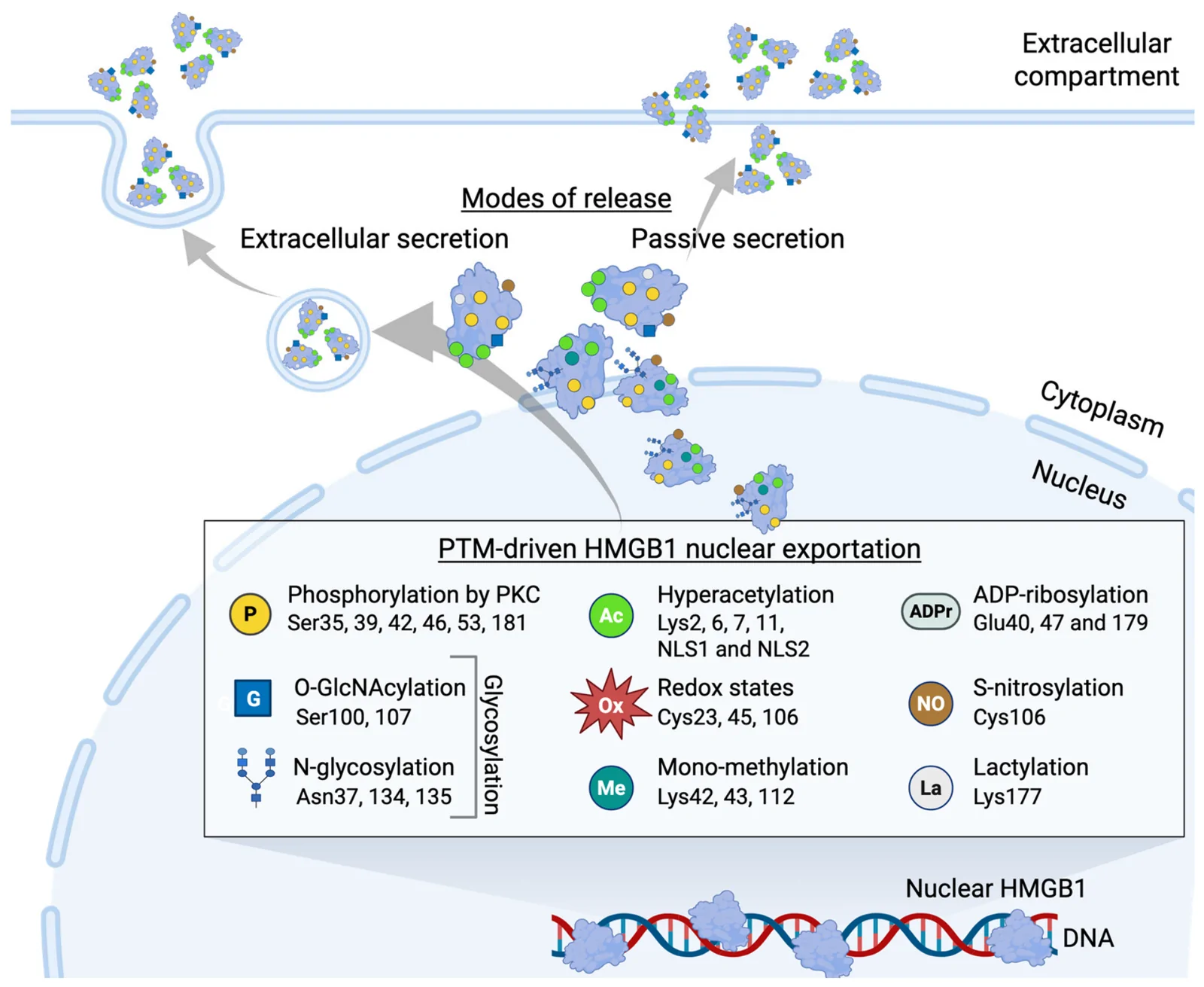
**Schematic of HMGB1 post-translational modifications (PTM)-driven translocation between cellular compartments.** Nuclear HMGB1 functions as a chromatin-associated protein involved in DNA organization, genome stability, and transcriptional regulation. Under conditions of cellular stress, inflammatory stimulation, or metabolic reprogramming, HMGB1 undergoes multiple post-translational modifications that regulate its subcellular localization and extracellular release. Representative PTMs include phosphorylation (Ser35, Ser39, Ser42, Ser46, Ser53, and Ser181), O-GlcNAcylation (Ser100 and Ser107), N-glycosylation (Asn37, Asn134, and Asn135), acetylation (Lys2, Lys6, Lys7, Lys11, NLS1, and NLS2), oxidation (Cys23, Cys45, and Cys106), mono-methylation (Lys42, Lys43, and Lys112), ADP-ribosylation (Glu40, Glu47, and Glu179), lactylation (Lys177), and S-nitrosylation (Cys106). These modifications promote HMGB1 nuclear export either individually or cooperatively, leading to its accumulation in the cytoplasm and eventual release into the extracellular compartment. Extracellular HMGB1 may arise through active secretion from viable cells or passive release from damaged or dying cells. Distinct PTMs can influence HMGB1 localization, secretion, molecular interactions, receptor engagement, and biological activity, thereby contributing to context-dependent responses within the tumour microenvironment. Abbreviations: P, phosphorylation; G, O-GlcNAc; Ac, acetylation; Ox, oxidation/redox modification; Me, methylation; ADPr, ADP-ribosylation; NO, S-nitrosylation; La, lactylation.

Under different microenvironmental conditions, such as cellular stress [26], HMGB1 is present in various post-translationally modified forms. PTMs represent chemical alterations of proteins during or after their synthesis through the reversible or irreversible addition or removal of functional groups. These modifications directly impact protein stability, subcellular localization, and functional activity, ultimately altering global biological processes and potentially driving disease progression [27,28]. The subcellular localization of HMGB1 is regulated by a diverse array of PTMs. Representative HMGB1 PTMs and their reported functions are summarized in Figure 2. Importantly, the biological activities of HMGB1 are determined not only by its expression level but also by its subcellular localization, mode of release, PTM status, redox state, and interactions with cognate receptors. These regulatory features collectively shape the functional consequences of HMGB1 signaling and may vary substantially across biological and disease contexts. Therefore, throughout this review, evidence is interpreted within the specific molecular state in which HMGB1 was examined whenever such information is available.

### 3.1. Phosphorylation

HMGB1 phosphorylation is largely regulated by classical protein kinase C (PKC) isoforms targeting regions around its nuclear localization sequences (NLS). HMGB1 is reported to be phosphorylated at multiple serine residues, specifically Ser35, Ser39, Ser42, Ser46, Ser53, and Ser181 [29], which collectively facilitate its translocation to the cytoplasm by weakening its DNA-binding affinity and disrupting its structural folding stability [30]. Following this hyperphosphorylation-dependent nuclear detachment, it is actively secreted by the cell via a calcium-dependent secretory mechanism [31].

### 3.2. Glycosylation

Glycosylation describes the biochemical process of covalently binding oligosaccharides, in the form of glycans, to specific amino acid residues on target proteins, with N- and O-glycosylation representing the two major subtypes commonly associated with human disease states [28]. This carbohydrate modification also serves as an important regulatory PTM for HMGB1.

#### 3.2.1. N-glycosylation

N-glycosylation of HMGB1 occurs selectively at the Asn37, Asn134, and Asn135 consensus motifs [32]. This specific sugar attachment has been found to enhance its baseline nuclear mobility and actively encourage its nucleocytoplasmic exportation. Furthermore, N-glycosylation contributes to HMGB1 stability by protecting it from ubiquitin-mediated proteasomal degradation.

#### 3.2.2. O-glycosylation

HMGB1 undergoes targeted O-GlcNAcylation, a type of O-glycosylation currently reported for HMGB1 [28,33]. O-GlcNAcylation is mediated by O-GlcNAc transferase (OGT) at Ser100 and Ser107, with Ser100 representing the predominant modification site affecting HMGB1 DNA-binding activity. This metabolic modification alters the charge distribution of the protein, which has been associated with reduced DNA-binding activity and may impair DNA repair-related functions [33]. 

### 3.3. Acetylation

Acetylation is a reversible covalent donation of an acetyl group by the metabolic cofactor acetyl-coenzyme A (acetyl-CoA) to either the N-terminus of proteins or specific internal lysine residues. The nuclear localization of HMGB1 is dictated by acetylation at its N-terminal region and its dual NLS domains, a process governed by host histone acetyltransferases (HATs) such as PCAF, CBP, and p300 [34]. Upon cellular stimulation or stress, hyperacetylation can be rapidly induced across six lysine residues [34]. The dense clustering of these acetyl groups neutralizes the positive electrostatic charge of the NLS domains, suppressing HMGB1’s affinity for genomic DNA and thereby promoting its nucleocytoplasmic translocation by impairing nuclear retention and facilitating cytoplasmic accumulation. Furthermore, robust acetylation within the N-terminal region, particularly at Lys2, Lys6, Lys7, and Lys11, has been associated with actively secreted HMGB1 [35]. 

### 3.4. Oxidation

During disease states, local tissue oxidation and redox levels are often altered, causing proteins to respond to environmental stressors such as severe hypoxia and the accumulation of reactive oxygen species (ROS). HMGB1 is a highly redox-sensitive protein that exists in several distinct functional redox isoforms: fully reduced HMGB1 (all-thiol), partially oxidized disulfide-HMGB1, terminally oxidized sulfonyl-HMGB1, and dimerized HMGB1, a higher-order HMGB1 species reported under oxidative conditions [36]. These redox modifications occur on three highly conserved, redox-active cysteine residues: Cys23, Cys45, and Cys106. In the partially oxidized disulfide form, HMGB1 contains an intramolecular disulfide bond between Cys23 and Cys45, while Cys106 remains in the reduced thiol state [37]. Under conditions of excessive oxidative stress, Cys23, Cys45, and Cys106 can undergo irreversible oxidation to sulfonyl (–SO3H) states, generating terminally oxidized HMGB1. Importantly, redox modifications not only influence HMGB1 secretion and extracellular localization but also help determine its receptor engagement and biological activity. For example, fully reduced HMGB1 primarily exerts chemotactic functions, whereas disulfide HMGB1 exhibits cytokine-inducing activity [38], while terminally oxidized HMGB1, in which the cysteine residues are oxidized to sulfonyl states, is generally considered biologically inactive [37].

### 3.5. Methylation

Methylation, a PTM characterized by the transfer of a methyl group onto lysine or arginine residues, is also well characterized in HMGB1. Like many other PTMs, mono-methylation of Lysine-42 (Lys42) induces a conformational shift within the A-box that severely weakens its DNA-binding affinity, resulting in its redistribution into the cytoplasm via passive diffusion out of the nucleus [39], a phenomenon that is well-documented in activated neutrophils. Similarly, mono-methylation at Lys112 has been found to contribute to its nucleocytoplasmic translocation [40]. Furthermore, Lys43 methylation has also been identified on HMGB1 and functions to disrupt its interaction with inflammation-related proteins, revealing its multiple roles in modulating the immune environment [41].

### 3.6. ADP-Ribosylation 

ADP-ribosylation is a post-translational modification involving the transfer of one or more ADP-ribose moieties from nicotinamide adenine dinucleotide (NAD^+^) to specific amino acid residues on target proteins [42]. In HMGB1, DNA alkylation-induced activation of poly(ADP-ribose) polymerase (PARP) promotes its translocation from the nucleus to the cytoplasm [43]. Interestingly, poly(ADP-ribosylation) of HMGB1 facilitates subsequent acetylation [44]. These PTMs act cooperatively to promote HMGB1 nuclear export.

### 3.7. Lactylation

Lactylation, a lactate-associated PTM, has also been reported on HMGB1 under hypoxic conditions [45]. Current evidence suggests that HMGB1 lactylation at Lys177 may be mediated through p300-dependent mechanisms, potentially involving lactyl-CoA as a donor substrate, thereby promoting HMGB1 nuclear export.

### 3.8. S-nitrosylation

HMGB1 has been reported to undergo S-nitrosylation, a modification involving the covalent binding of nitric oxide (NO) to cysteine thiols by inducible nitric oxide synthase (iNOS)-derived NO, thereby promoting inflammation-induced HMGB1 secretion [46] and sustaining a proinflammatory environment in disease states.

Collectively, these findings suggest that HMGB1 PTMs do not function in isolation. Instead, multiple modifications may interact cooperatively or sequentially to regulate HMGB1 localization, secretion, receptor engagement, and biological activity.

A summary of HMGB1 post-translational modifications, modification sites, and reported biological effects is provided in Table 1.

## 4. Redox-Dependent Receptor Engagement and Emerging Roles of HMGB1 PTMs

Apart from regulating nucleocytoplasmic transport and extracellular accumulation, certain HMGB1 molecular states may further influence interactions between secreted HMGB1 and its surface receptors through alterations in protein conformation and molecular interactions. Among these, HMGB1 redox state represents one of the best-characterized determinants of receptor selectivity and downstream signaling. The principal extracellular receptors involved in HMGB1 signaling include Toll-like receptors (TLRs), particularly TLR2 and TLR4, and RAGE and CXCR4 through formation of an HMGB1–CXCL12 heterocomplex [49,50,51,52]. Fully reduced HMGB1 (frHMGB1) forms a heterocomplex with CXCL12, which subsequently signals through CXCR4. In contrast, disulfide HMGB1 (dsHMGB1), characterized by a disulfide bond between Cys23 and Cys45 with Cys106 remaining reduced, loses its ability to participate in CXCL12-CXCR4 signaling and instead interacts with the TLR4/MD-2 complex to induce pro-inflammatory cytokine production [38]. Conversely, terminally oxidized (sulfonyl) HMGB1 is generally considered biologically inactive [37,53]. Although HMGB1 redox state represents an important determinant of receptor engagement, receptor selectivity is also influenced by factors such as molecular binding partners, oligomeric state, receptor/co-receptor expression, cell type, and local microenvironmental conditions. Therefore, receptor selectivity should be viewed as a context-dependent process rather than a strictly binary consequence of HMGB1 redox status.

Although direct evidence linking PTMs other than redox modifications to receptor-specific interactions remains limited, many PTMs regulate HMGB1 localization, secretion, and extracellular availability, thereby potentially influencing receptor accessibility and signaling outcomes. Redox regulation currently provides the clearest example of how distinct molecular states of HMGB1 can generate divergent biological responses through differential receptor engagement. These observations provide a framework for understanding how PTM-dependent regulation may contribute to context-specific HMGB1 signaling within the TME.

## 5. HMGB1–Receptor Interaction in Intercellular Communication in the TME

The PTM-dependent regulation of macrophage polarization highlights how extracellular HMGB1 coordinates intercellular crosstalk within the immune landscape. While direct investigations into how HMGB1 modulates immune cells within NPC remain ongoing, evidence from other malignancies demonstrates its strong capacity to interact with surrounding cell populations, primarily through TLRs and RAGE. This cytokine-driven mode of microenvironmental remodeling parallels our previous review regarding midkine (MK) signaling, where complex soluble factor networks similarly govern dysregulated immune cell infiltration and intercellular communication within the NPC niche [54]. Notably, TLRs are highly expressed in NPC tissues, with TLR2 and TLR5 expression directly correlating with patient viral status [55]. Consequently, the HMGB1–receptor signaling axis may function as an important communication network that contributes to immune-cell crosstalk within the NPC TME.

### 5.1. Innate Immunity: Context-Dependent Macrophage Polarization and Myeloid-Derived Suppressor Cell Expansion

#### 5.1.1. Macrophage Polarization and Biological Heterogeneity

The capacity of extracellular HMGB1 to drive divergent macrophage phenotypes, ranging from pro-inflammatory M1-like states to immunosuppressive M2-like states, remains a subject of ongoing debate. For instance, HMGB1 has been reported to promote M1-like polarization in oral squamous cell carcinoma [56], whereas other studies demonstrate that extracellular HMGB1 preferentially induces M2-like macrophage polarization via RAGE signaling in osteosarcoma [57] and TLR2 in hepatocellular carcinoma [58]. Rather than representing true contradictions, these apparently conflicting observations likely reflect biological and methodological heterogeneity across studies. Several non-mutually exclusive factors may account for these discrepancies, including differences in HMGB1 redox and PTM states, receptor engagement, tumor-specific microenvironmental conditions, and macrophage classification strategies.

First, distinct HMGB1 isoforms generated through redox modifications and other PTMs possess different biological activities. Second, HMGB1 can engage multiple receptors, including TLR2, TLR4, and RAGE, which activate distinct downstream signaling pathways. Third, macrophage responses are strongly influenced by tissue-specific and tumor-specific microenvironmental conditions, including hypoxia, oxidative stress, metabolic reprogramming, and viral infection status. Finally, differences in experimental design and macrophage classification strategies may contribute to inconsistent interpretations of polarization states across studies.

For example, disulfide-HMGB1 preferentially activates the TLR4 pathway and induces the production of pro-inflammatory cytokines, including interleukin (IL)-6 [38,51,59,60], whereas fully reduced HMGB1 exhibits distinct biological properties and does not directly promote classical M1-like polarization. In addition, the apparently conflicting effects of HMGB1 on macrophage polarization may partly reflect limitations of the traditional M1/M2 classification framework. Although many studies categorize macrophages using a restricted set of markers, recent single-cell and spatial transcriptomic analyses have revealed multiple transcriptionally and functionally distinct macrophage populations that exist along a continuum of activation states rather than as discrete M1 or M2 subsets. Consequently, macrophage populations classified differently across studies may represent overlapping, transitional, or context-dependent states rather than fundamentally opposing phenotypes. Factors such as HMGB1 molecular state, receptor engagement, ligand concentration, mode of delivery, and local TME may further influence these responses. Future single-cell and spatial profiling studies will therefore be required to more precisely define HMGB1-responsive macrophage subsets in NPC.

Tumor-specific microenvironmental factors may further contribute to divergent observations across cancer types. In NPC, chronic EBV infection establishes a unique inflammatory and oxidative microenvironment that differs substantially from many non-viral malignancies. For example, EBNA1 has been shown to increase intracellular ROS through upregulation of the NADPH oxidases NOX1 and NOX2 [13]. Such an oxidative environment may influence HMGB1 signaling and macrophage functional states. For example, HMGB1 has been reported to promote autophagy and NF-κB p65 downregulation in specific contexts, processes associated with M2-like polarization [58]. However, direct evidence demonstrating that EBV-driven oxidative stress determines HMGB1-dependent macrophage polarization remains limited. 

#### 5.1.2. Potential Links Between HMGB1 and Tumor-Associated Macrophages

Recent single-cell transcriptomic studies have further revealed substantial heterogeneity within the TAM population in NPC, including the enrichment of a distinct C1q^+^ TAM subpopulation [61]. This subset has been associated with tumor progression, recurrence, and poor patient survival [62]. Functionally, C1q^+^ TAMs serve as a major source of complement component C1q within the TME and have been implicated in immune suppression through the promotion of inhibitory receptor expression on cytotoxic CD8^+^ T cells and the development of T-cell dysfunction [63]. Emerging evidence further suggests that C1q can modulate T-cell metabolic programming, thereby limiting effector T-cell activity [64]. Importantly, several of the immunological outcomes associated with C1q^+^ TAMs resemble biological effects previously attributed to HMGB1 signaling, including immune suppression, T-cell dysfunction, and metabolic reprogramming. Given the established role of HMGB1-RAGE/TLR signaling in macrophage activation and transcriptional reprogramming, it is conceivable that HMGB1 may influence the emergence or function of specialized TAM subsets, including C1q^+^ macrophages. However, direct evidence supporting such a relationship in NPC is currently lacking. We therefore propose a hypothetical model in which HMGB1-mediated macrophage reprogramming and C1q-associated macrophage pathways may act in parallel or partially overlapping immunosuppressive networks within the NPC microenvironment. At present, whether HMGB1 directly regulates C1q expression, promotes the development of C1q^+^ TAMs, or cooperates with C1q-dependent mechanisms to drive T-cell dysfunction remains unknown. These possibilities should therefore be regarded as hypothesis-generating concepts that require future experimental validation.

#### 5.1.3. Exosomal HMGB1 and Metabolic Regulation of Macrophage Function

In addition to soluble protein release, gastric cancer cells actively secrete HMGB1-enriched exosomes that promote macrophage reprogramming toward an M2-like TAM phenotype through inhibition of NF-κB signaling [65]. These macrophages subsequently secrete pro-tumorigenic factors that enhance cancer cell migration and invasion while further stimulating HMGB1 expression, thereby establishing a positive feedback loop [58]. Although direct evidence in NPC is currently lacking, these findings suggest a potential mechanism by which tumor-derived exosomal HMGB1 could contribute to macrophage reprogramming within the TME.

Whether a similar exosomal HMGB1-mediated regulatory circuit exists in EBV-associated NPC remains unknown. However, the highly glycolytic and lactate-rich microenvironment characteristic of NPC [66] may provide conditions that favor HMGB1 secretion and functional diversification. In other experimental systems, lactate exposure has been shown to stimulate macrophage-derived HMGB1 release [67], and has been associated with PTMs including lactylation, hyperacetylation [68], and phosphorylation [69]. These observations raise the possibility that metabolically regulated HMGB1 species may contribute to immune modulation in NPC. Nevertheless, the presence, PTM composition, and biological functions of exosomal HMGB1 in NPC have not yet been directly established and require further investigation.

#### 5.1.4. HMGB1-Mediated Regulation of Myeloid-Derived Suppressor Cells

In parallel, myeloid-derived suppressor cells (MDSCs) represent another key immunosuppressive population shaped by HMGB1. Li et al. were the first to report that secreted HMGB1 facilitates the recruitment of MDSCs [70]. Mechanistically, extracellular HMGB1 acts as a high-affinity ligand for RAGE and TLR4 expressed on MDSCs [71,72]. Upon receptor engagement, Parker et al. further demonstrated that HMGB1 activates MDSCs via NF-κB signaling, promotes their differentiation from bone marrow progenitors, and enhances their immunosuppressive activity, including increased hydrogen peroxide (H_2_O_2_) production. Furthermore, HMGB1 augments MDSC–macrophage crosstalk through IL-10 secretion and impairs T cell homing by sustaining ADAM17 expression, leading to downregulation of L-selectin on naïve T cells [72]. This HMGB1-dependent myeloid dysregulation aligns closely with the EBV-driven pathogenesis of NPC. MDSCs are markedly expanded in NPC and correlate with tumor progression and poor clinical outcomes [73]. These cells exhibit notable metabolic plasticity, utilizing both glycolysis and oxidative phosphorylation to support their expansion and suppressive function [12]. Consistently, EBV–LMP1–driven extramitochondrial glycolysis promotes IL-6 and GM-CSF production via p-p65, COX-2, and NLRP3 signaling, thereby facilitating MDSC expansion within the TME and reinforcing immunosuppression in NPC [12].

### 5.2. Adaptive Immunity: T Cells and B Cells

#### 5.2.1. T Cells

Despite substantial leukocyte infiltration, NPC exhibits features of both immune activation and immune suppression. It is generally considered an immunologically “hot” tumor, with transcriptomic analyses indicating that over 80% of tumor-infiltrating immune cells are B and T lymphocytes [74]. Despite this extensive immune infiltration, local T-cell activity remains functionally impaired within the TME. Recent findings by Xie et al. suggest that epitranscriptomic regulation in NPC epithelial cells may contribute to this immunological imbalance [75]. Specifically, the RNA acetyltransferase NAT10 stabilizes DDX5 mRNA through ac4C (N4-acetylcytidine) modification, thereby enhancing HMGB1 expression and extracellular release from NPC cells. The NAT10/DDX5/HMGB1 axis has been associated with suppression of both CD4^+^ and CD8^+^ T-cell effector functions, supporting a potential role in tumor-associated immunosuppression.

A major hallmark contributing to T-cell suppression within the NPC TME is the dense infiltration of CD4+CD25+FOXP3+ regulatory T cells (Tregs), the abundance of which correlates with elevated circulating EBV DNA copies [76]. Intriguingly, a similar clinical pattern has been reported: HMGB1 expression is elevated in EBV-positive NPC and positively correlates with LMP1 expression levels [6]. Given that NPC-derived exosomes can regulate Treg recruitment, expansion, and suppressive activity [77], these observations suggest a potential intersection between EBV signaling, HMGB1 biology, and Treg-mediated immunosuppression. One possible model is that EBV/LMP1-induced HMGB1 upregulation contributes not only to tumor cell survival but also to immune remodeling within the NPC microenvironment. If HMGB1 is incorporated into tumor-derived exosomes, as reported in other cancer types, exosomal HMGB1 could potentially influence Treg recruitment or function through receptor-mediated signaling pathways involving RAGE, TLR4, or related mechanisms. However, whether HMGB1-containing exosomes are present in NPC, whether they directly regulate Tregs, and whether RAGE/TLR4-dependent signaling mediates these effects remain unknown. Collectively, these observations raise the possibility that EBV-driven HMGB1 signaling may contribute to Treg-associated immunosuppression in NPC. However, the existence of a complete HMGB1-mediated exosome-RAGE/TLR4-Treg signaling axis has not been experimentally demonstrated and should therefore be regarded as a hypothesis requiring future validation.

Concurrently, NPC tumors subvert remaining effector T cells through activation of immune checkpoint pathways. NPC tissues frequently exhibit upregulation of the immune co-inhibitory receptor TIM-3 on infiltrating T lymphocytes, consistent with an exhausted phenotype [78]. Beyond its role as an exhaustion marker, TIM-3 has also been identified as a receptor for HMGB1 in other biological contexts, where HMGB1-TIM-3 interactions can modulate immune responses and nucleic acid sensing. In ovarian cancer, HMGB1-TIM-3 signaling has been linked to IL-32 regulation and T-cell dysfunction [79]. However, whether a similar HMGB1-TIM-3-IL-32 axis operates in NPC remains to be determined. In parallel, Galectin-9—another key ligand for TIM-3—exhibits distinct spatial proximity to CD8^+^ T cells within the NPC TME [80]. This spatially restricted Galectin-9 signaling axis has been implicated in immune regulation and T-cell dysfunction. Together, these observations suggest that TIM-3-associated signaling pathways may contribute to T-cell exhaustion within the NPC microenvironment. However, the relative contributions of HMGB1, Galectin-9, and other TIM-3 ligands, as well as the existence of a functional HMGB1-TIM-3-IL-32 signaling axis in NPC, remain incompletely defined and require further investigation.

#### 5.2.2. B Cells

B-cell enrichment is frequently observed within tertiary lymphoid structures (TLS), which are ectopic lymphoid aggregates characterized by dense B-cell zones. High TLS density and structural integrity are strongly associated with favorable clinical outcomes [81]. Conversely, Chen et al. demonstrated that a low-TLS B-cell signature is associated with poor prognosis. The functional state of extracellular HMGB1 may influence B-cell organization within the TME. Fully reduced HMGB1 can form a heterocomplex with CXCL12 to regulate B-cell trafficking [82]. Given the importance of coordinated B-cell recruitment in TLS development, it is tempting to speculate that similar mechanisms may support immune-cell aggregation and TLS formation in NPC. However, direct evidence for such a role in NPC is currently lacking.

In contrast, progression toward a hypoxic and oxidatively stressed microenvironment may shift HMGB1 toward the disulfide form [38]. Unlike the fully reduced form, disulfide HMGB1 preferentially signals through TLR pathways and promotes pro-inflammatory responses rather than chemokine-directed cellular organization. This shift in HMGB1 signaling raises the possibility that distinct redox states may influence B-cell behavior differently within the TME.

Consistent with this concept, advanced NPC has been associated with the accumulation of a CD86^+^ memory B-cell subset linked to poor clinical outcomes [83]. Given that co-stimulation via TLR2 and CD86 can enhance B-cell survival [84], it is plausible that disulfide HMGB1 may act as a microenvironmental factor sustaining this potentially tumor-promoting B-cell population. Nevertheless, direct mechanistic evidence in NPC is currently unavailable. 

Furthermore, regulatory B cells (Bregs) represent another immunosuppressive subset enriched in the hypoxia-driven NPC microenvironment via mitogen-activated protein kinase (MAPK) pathway activation [85]. Tumor-derived exosomal HMGB1 in hepatocellular carcinoma promotes Breg expansion through TLR2/4-dependent MAPK signaling [86]. These Bregs subsequently secrete IL-10, which is associated with impaired CD8+ T cell cytotoxicity. Whether this mechanism is conserved in NPC remains to be determined. Together, these findings suggest that distinct HMGB1 molecular states may differentially influence B-cell behavior within the NPC microenvironment. However, whether specific HMGB1 PTM or redox states directly regulate TLS formation, memory B-cell maintenance, or regulatory B-cell development in NPC remains unknown and requires experimental validation.

In addition to immunoregulatory functions, extracellular HMGB1 may also contribute to functional reprogramming of tumor-associated B cells. HMGB1-enriched tumor niches promote a proliferative and pro-angiogenic B-cell phenotype characterized by increased secretion of vascular endothelial growth factor (VEGF) [87]. This shift may contribute to angiogenic remodeling of the hypoxic TME, thereby supporting tumor growth, invasion, and metastasis.

**Figure 3:**
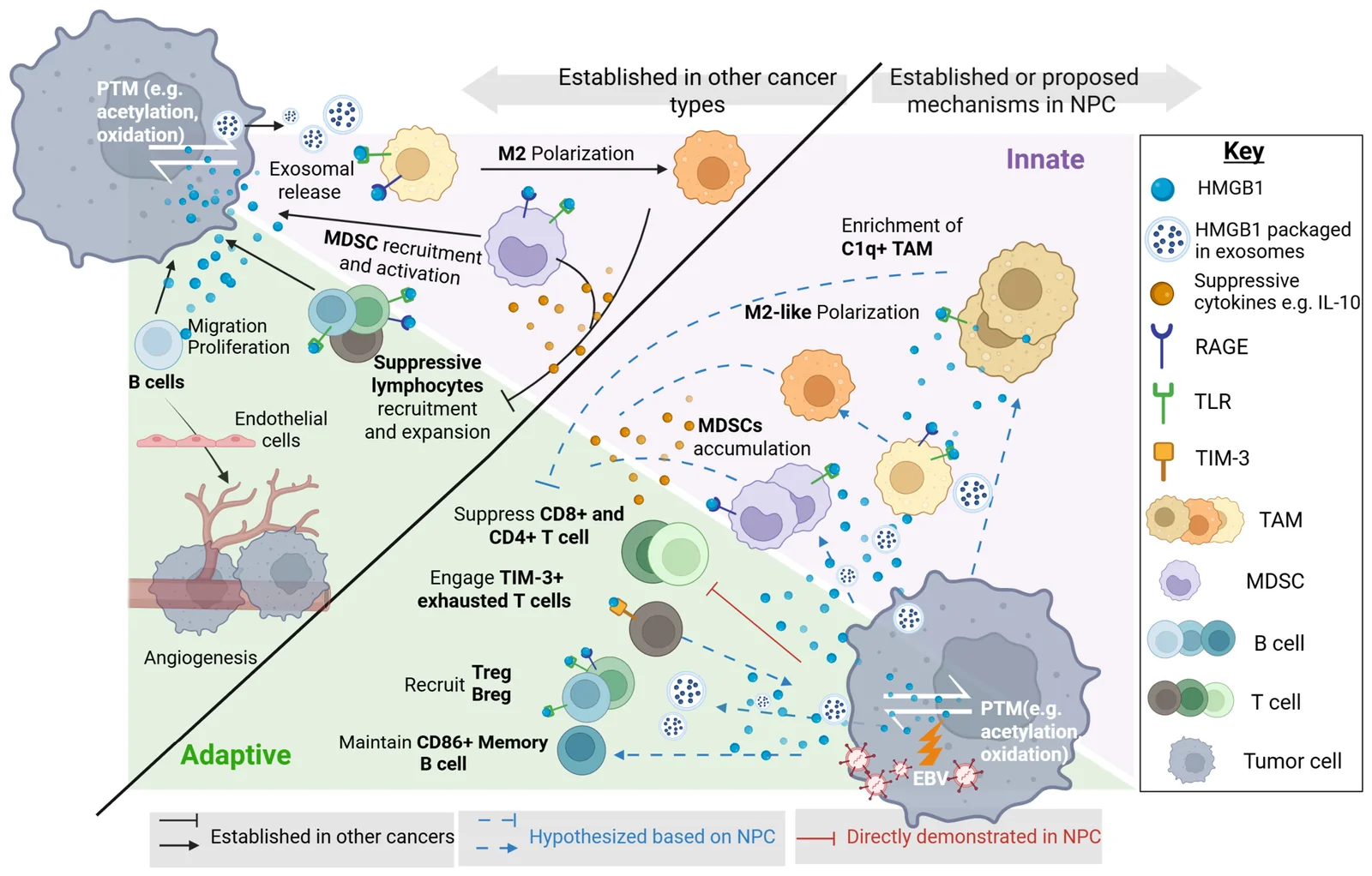
**A conceptual framework of HMGB1-mediated immune regulation in the nasopharyngeal carcinoma (NPC) tumour microenvironment (TME).** Compartmental Organization: The schematic uses a diagonal axis to distinguish pathways supported primarily by evidence from non-NPC cancer models (top-left) from mechanisms that have been directly demonstrated, partially supported, or proposed in NPC (bottom-right). Background shading delineates Innate Immunity (light-purple shaded) and Adaptive Immunity (green-shaded) compartments. EBV-Associated Regulation of HMGB1: EBV infection in NPC has been associated with signalling pathways that may influence host post-translational modifications (PTMs), including acetylation and oxidation, potentially affecting HMGB1 intracellular trafficking, exosomal packaging, and extracellular release. Although several EBV-driven mechanisms have been proposed, direct evidence linking specific HMGB1 PTMs to EBV-associated NPC remains limited. Innate Immune Remodeling: Extracellular and exosomal HMGB1 [65] have been reported to regulate macrophage polarization [57,58] and myeloid-derived suppressor cell (MDSC) expansion in multiple cancer models [72,73] through RAGE- and TLR-dependent signalling pathways. Emerging evidence suggests that HMGB1 may contribute to immunosuppressive myeloid networks in NPC, including pathways potentially associated with C1q^+^ tumour-associated macrophages (TAMs) [61], although direct mechanistic evidence remains limited. Adaptive Immune Regulation: HMGB1-mediated signalling has been associated with impaired effector T-cell activity in NPC [75] and has been proposed to influence additional adaptive immune processes, including TIM-3-associated pathways [78], regulatory T-cell (Treg) [77] and regulatory B-cell (Breg) [84] populations, and memory B-cell dynamics [83]. However, many of these mechanisms are derived from studies in other cancer types and require experimental validation in NPC [79,86,87,88]. Symbol and Pathway Key: Solid black arrows indicate relationships experimentally validated in non-NPC cancer models. Dashed blue arrows indicate hypothesized or inferred interactions based on NPC biology and evidence from related systems. Red arrows indicate pathways directly demonstrated in NPC. Molecular symbols represent soluble HMGB1 (blue spheres), exosomal HMGB1 (vesicular particles), suppressive cytokines (orange spheres), RAGE (blue receptors), TLRs (green receptors), and TIM-3 (orange receptors). Reference numbers correspond to the primary studies supporting each pathway.

In summary, HMGB1 has emerged as an important regulator of immune responses within the NPC microenvironment, with reported roles in myeloid-cell function, regulatory immune-cell recruitment, and lymphocyte dysfunction. Emerging evidence further suggests that distinct HMGB1 molecular states, shaped by PTMs, redox status, and mode of release, may differentially influence these immune processes and contribute to context-dependent immune regulation. By integrating findings from NPC and related biological systems, a framework emerges in which HMGB1 participates in complex intercellular communication networks linking innate and adaptive immunity. However, many of the proposed mechanisms remain incompletely characterized in NPC and require further experimental validation. A summary of the HMGB1–receptor interactions discussed in this section is provided in Table 2. Collectively, these observations highlight the multifaceted and context-dependent functions of extracellular and exosomal HMGB1 and support further investigation into how HMGB1 molecular states influence immune regulation within the NPC TME (Figure 3).

## 6. Therapeutic Implications in NPC 

### 6.1. HMGB1 as a Potential Therapeutic Target

In addition to its overexpression, HMGB1 holds clinical relevance in NPC. Elevated HMGB1 levels in patient tissues correlate positively with TNM stage and overall disease progression. Clinically, high HMGB1 expression is associated with poor prognosis, including reduced overall survival (OS) and progression-free survival (PFS) [7]. While these observations support a clinically relevant association between HMGB1 expression and NPC progression, the available evidence is derived primarily from retrospective cohorts and requires further validation in larger patient populations treated with current standards of care. Paradoxically, extracellular HMGB1 release may also be induced by conventional treatment modalities used in NPC, including radiotherapy, chemotherapy, and emerging anti-cancer agents. The following sections outline how standard therapies induce secondary HMGB1 release within the NPC microenvironment and discuss the context-dependent consequences of treatment-associated HMGB1 signaling.

#### 6.1.1. Radiotherapy

As a cornerstone treatment modality for NPC, high-dose precision radiotherapy is utilized to induce severe genomic DNA damage and directly drive cancer cell death [89]. Baseline HMGB1 expression has been found to correlate significantly with initial radiotherapy responses in NPC patients [7], and its inhibition successfully overcomes resistance to both radio- and chemotherapy by reducing DNA repair efficiency in surviving cells [90]. However, radiation-damaged, dying cells also actively release HMGB1 into the extracellular space [91]. Depending on its molecular state, concentration, timing, and immune context, treatment-induced HMGB1 may exert both beneficial and detrimental effects. On the other hand, persistent HMGB1 signaling has been associated with tumor-cell survival, tissue repair responses, chronic inflammation, and treatment resistance. Therefore, the therapeutic implications of radiation-induced HMGB1 release remain context-dependent and require further investigation.

#### 6.1.2. Chemotherapy and Anti-Cancer Agents

Systemic chemotherapy is routinely administered with or following radiotherapy to consolidate treatment outcomes in NPC patients. Microtubule inhibitors commonly used in these regimens, such as docetaxel and paclitaxel [92], have been reported to promote HMGB1 release [93]. Furthermore, additional therapeutic strategies are being explored for NPC, including the combination of decitabine with radiotherapy to enhance radiosensitivity [94] and recent phase II clinical trials evaluating oral azacitidine in advanced metastatic NPC [95]. Both decitabine and azacitidine function as epigenetic modifiers that covalently and irreversibly bind DNMTs to induce cell death [96]. However, these agents have also been associated with increased HMGB1 release [93]. Given the growing use of multimodal treatment strategies, understanding the biological consequences of treatment-associated HMGB1 release may be important for improving therapeutic responses and overcoming potential resistance mechanisms.

Collectively, these observations suggest that HMGB1 upregulation is not only a hallmark of NPC progression but also a shared consequence of current therapeutic interventions, highlighting its potential contribution to treatment resistance.

#### 6.1.3. Therapeutic Implications of Immunogenic Cell Death and the Dual Role of HMGB1

Although extracellular HMGB1 is frequently associated with chronic inflammation, immune suppression, and treatment resistance, complete inhibition of HMGB1 signaling may not always be therapeutically desirable. HMGB1 released from dying tumour cells following radiotherapy and chemotherapy represents a key DAMP involved in immunogenic cell death (ICD) that is characterized by the induction of T-cell-mediated anticancer immune responses. In this context, extracellular HMGB1 engages TLR4 on dendritic cells, facilitating antigen processing and cross-presentation, thereby promoting anti-tumour T-cell priming and adaptive immune responses [97]. Thus, treatment-induced HMGB1 release may contribute to the efficacy of conventional therapies rather than solely promoting tumour progression [98]. Consequently, future therapeutic strategies may require selective modulation of pathogenic HMGB1 signaling rather than indiscriminate suppression of all HMGB1 activity. Preserving beneficial ICD-associated functions while limiting chronic HMGB1-driven immune dysregulation remains an important translational challenge in NPC.

### 6.2. RNA Acetylation–Mediated Regulation of HMGB1 in NPC 

Recent advances in epitranscriptomic regulation further expand the oncogenic network surrounding HMGB1 in NPC. As briefly mentioned in Section 5.2.1, the study by Xie et al. identified the acetyltransferase NAT10 as an upstream regulator of HMGB1 through N4-acetylcytidine (ac4C) RNA modification [75]. Mechanistically, NAT10-mediated ac4C modification enhances the stability and translational efficiency of transcripts such as DDX5, an RNA helicase, which in turn promotes HMGB1 expression. Elevated HMGB1 has been associated with immunosuppressive remodeling of the TME, including impaired antigen presentation and T-cell exhaustion [99,100]. Importantly, NAT10 activity was shown to directly contribute to resistance to anti–PD-1 therapy in NPC, while its inhibition restores CD4^+^ and CD8^+^ T cell functionality and sensitizes tumors to immune checkpoint blockade. Unlike acetylation, phosphorylation, or redox modifications that directly alter HMGB1 protein activity, the NAT10–ac4C–DDX5 axis acts upstream at the epitranscriptomic level to regulate HMGB1 expression. Thus, this mechanism represents a distinct layer of HMGB1 regulation rather than a PTM of the HMGB1 protein itself. These findings further emphasize that HMGB1 can be regulated at multiple molecular levels and may represent a therapeutically relevant target in NPC.

### 6.3. Current Landscape of HMGB1-Targeted Intervention

Given its association with unfavorable clinical outcomes and immunosuppressive remodeling of the TME, substantial research efforts have focused on targeting extracellular HMGB1. However, because HMGB1 may also contribute to ICD and anti-tumour immune activation under specific conditions, therapeutic intervention must balance suppression of pathogenic HMGB1 signaling with preservation of beneficial immune functions. Current HMGB1-targeted strategies therefore aim to selectively modulate disease-promoting HMGB1 activity rather than indiscriminately eliminate all HMGB1 signaling. HMGB1. Although HMGB1-targeted interventions in oncology remain relatively limited, particularly in NPC (Table 3), current strategies generally fall into two main categories: direct extracellular neutralization and inhibition of HMGB1 release. Understanding their distinct mechanisms of action is important for evaluating their potential therapeutic applications in NPC. 

#### 6.3.1. Extracellular Neutralization

Glycyrrhizin (GL), a bioactive triterpenoid derived from traditional Chinese licorice, is one of the most widely studied direct inhibitors of HMGB1. Structurally, GL binds to both the Box-A and Box-B domains of HMGB1 [103], thereby neutralizing its extracellular chemoattractant and mitogenic activities and disrupting its interaction with receptors such as TLR4 and RAGE [104]. HMGB1 has been implicated in DNA damage repair through interactions with components of the non-homologous end joining (NHEJ) machinery, including Ku70. In colorectal cancer models, GL was reported to enhance radiosensitivity by disrupting HMGB1-associated DNA repair pathways and impairing NHEJ activity [105]. However, this mechanism has not yet been directly validated in NPC. Nevertheless, these findings raise the possibility that HMGB1 inhibition may similarly influence treatment responsiveness in NPC and warrants further investigation. This possibility is particularly relevant because radiotherapy and platinum-based chemotherapy remain the mainstay treatments for NPC, and treatment resistance is closely associated with efficient DNA repair capacity. Beyond its biochemical binding, accumulating evidence demonstrates that GL can functionally suppress HMGB1-driven oncogenic signaling across multiple cancer types. 

In addition to its direct targeting of HMGB1, GL inhibits autophagy-dependent HMGB1 release from stromal cells such as cancer-associated fibroblasts (CAFs), thereby attenuating NF-κB-driven inflammatory signaling and invasive behavior [106]. Given that CAFs induce the formation of radioresistance and promote NPC cell survival [107], this ability to suppress HMGB1-mediated paracrine signaling may help disrupt tumor–stroma crosstalk that sustains immune evasion and tumor progression. Importantly, although glycyrrhizin is widely used as an HMGB1-binding compound, its biological effects are not restricted to HMGB1 neutralization. GL has been reported to modulate multiple inflammatory and stress-response pathways, making it difficult to attribute all observed antitumor effects exclusively to HMGB1 inhibition.

Other natural compounds targeting HMGB1 extracellular activity have also been described, including steroid derivatives from the Chinese herb Danshen and epigallocatechin gallate (EGCG), the primary polyphenolic component of green tea. Both have demonstrated activity in attenuating HMGB1-related pathological processes [108]. However, their mechanistic validation in NPC specifically remains limited compared to GL.

Limitations/drawbacks: Relying solely on extracellular neutralization is unlikely to fully eliminate the HMGB-driven tumor-promoting effects in NPC. First, because HMGB1 is a highly pleiotropic protein with vital physiological roles in nuclear DNA repair and homeostatic cell survival, prolonged systemic inhibition may lead to undesirable toxicities and the impairment of normal tissue homeostasis. Second, the chronically hypoxic and stress-adapted NPC microenvironment, together with persistent EBV lytic cycling, sustains continuous intracellular HMGB1 expression and active secretion. Thus, extracellular blockade primarily targets downstream signaling without shutting off the continuous intracellular source of HMGB1. Moreover, definitive evidence demonstrating target engagement, intratumoral drug exposure, and selective inhibition of extracellular versus nuclear HMGB1 in NPC remains limited.

Furthermore, the majority of evidence supporting HMGB1-neutralizing agents originates from acute inflammatory models, such as sepsis or ischemic injury, rather than the chronic, immunosuppressive, and spatially complex TME observed in NPC. This discrepancy raises concerns regarding the durability and clinical translatability of such approaches [108]. Collectively, these limitations highlight the need for combinatorial or upstream strategies, such as targeting HMGB1 release mechanisms, PTMs or transcriptional regulation, to achieve more effective and sustained therapeutic responses in NPC.

#### 6.3.2. Blockade of Interaction Through RAGE Receptor

As previously discussed, the HMGB1-RAGE axis represents a major signaling pathway linking extracellular HMGB1 to immunosuppressive populations within the TME. Consequently, RAGE inhibition has emerged as a therapeutic strategy to diminish tumor dependency on this axis. For instance, RAGE Antagonist Peptide (RAP) competitively binds to the extracellular domain of RAGE, successfully disrupting the ligand–receptor interaction [109]. Similarly, FPS-ZM1, a small-molecule inhibitor targeting the immunoglobulin-like V domain of RAGE, serves as another promising candidate to interfere with this signaling cascade [110]. 

Notably, RAGE is a multiligand receptor that also interacts with members of the S100 family, a group of calcium-binding proteins that can function as extracellular signaling molecules in a cytokine-like manner [111]. Among these, S100A14 has been implicated in modulating RAGE signaling in a context-dependent manner. While S100A14 can activate MAPK and NF-κB pathways in certain cancers [112], evidence in NPC suggests a more nuanced role. In vitro and in vivo studies demonstrate that S100A14 suppresses NPC cellular motility through modulation of NF-κB signaling, and its overexpression is associated with increased sensitivity to cisplatin [113]. These findings highlight that RAGE signaling in NPC is not uniformly tumor-promoting but instead reflects a dynamic balance between distinct ligands with divergent functional outcomes.

Limitations/drawbacks: Despite strong mechanistic rationale, targeting RAGE alone presents several limitations in the context of NPC. First, RAGE is widely expressed and plays important roles in normal physiology. Notably, it is highly expressed in lung tissue, where it is required for maintaining tissue homeostasis [114]. Second, the promiscuity of RAGE ligands, including HMGB1 and S100 family proteins, with potentially opposing functions introduces signaling redundancy and context-dependent effects. S100A14 may partially counteract HMGB1-driven oncogenic signaling, further underscoring the complexity of targeting the HMGB1–RAGE axis in NPC. As such, global RAGE inhibition may inadvertently suppress both tumor-promoting and tumor-restraining signals, limiting its therapeutic precision. Collectively, these challenges suggest that RAGE-targeted strategies are unlikely to be effective as standalone therapies in NPC and will likely require integration into upstream or combinatorial targeting strategies.

#### 6.3.3. Disruption of Release

In contrast to receptor blockade, a more upstream strategy involves preventing the release of HMGB1 itself. This process is tightly regulated by PTMs, as discussed above, where acetylation, phosphorylation, and redox changes govern HMGB1 nucleocytoplasmic translocation and secretion. Targeting these PTM-dependent mechanisms therefore represents one potential approach to limiting the availability of extracellular HMGB1.

Ethyl Pyruvate (EP), an aliphatic ester of pyruvic acid, is a well-characterized inhibitor of HMGB1 release [115]. EP has been reported to suppress tumor growth in multiple malignancies by targeting the HMGB1–RAGE axis and downstream signaling pathways, including serine/threonine kinase (AKT), NF-κB and STAT3 [116]. Mechanistically, EP acts in part by influencing the PTM landscape of HMGB1. Specifically, EP suppresses HMGB1 phosphorylation, potentially through calcium chelation [117], and inhibits hyperacetylation, a PTM associated with HMGB1 nuclear export [118]. By limiting these PTM-driven changes in HMGB1 localization, EP promotes nuclear retention of HMGB1 and reduces its extracellular accumulation. Consequently, EP may reduce extracellular HMGB1 signaling by limiting its release from cells.

The ability of EP to inhibit HMGB1 release has been validated in mesothelioma models, where treatment resulted in nuclear accumulation of HMGB1 accompanied by a reduction in extracellular levels [119]. Beyond regulating HMGB1 localization, EP has also been shown to overcome therapy resistance by shifting cell death from pro-inflammatory necrosis toward apoptosis and by disrupting cytoprotective autophagy [120,121]. This mechanism is particularly relevant in NPC, where chronic EBV-associated stress promotes HMGB1 release and sustains pro-survival signaling. We have previously demonstrated that NPC-derived galectin-9 promotes autophagy while restricting necrotic cell death [80]. In this context, the dual function of EP, reducing HMGB1 release while simultaneously disrupting autophagy, may be especially effective in limiting tumor survival and breaking HMGB1-driven feedforward signaling loops. It should be noted that EP exerts pleiotropic biological effects beyond HMGB1 regulation, including modulation of inflammatory signaling, oxidative stress responses, and cellular metabolism, which may contribute to its observed anti-tumor activities.

Collectively, these agents represent a heterogeneous group of HMGB1-modulating approaches, including direct HMGB1 neutralizers, receptor antagonists, and inhibitors of HMGB1 release, rather than a unified therapeutic platform. 

## 7. Current Controversies and Unresolved Questions

### 7.1. Distinguishing Established Evidence from Emerging Concepts in NPC

Despite increasing interest in HMGB1 biology in NPC, direct mechanistic evidence remains limited for several aspects of its post-translational regulation, receptor-mediated signaling, and immune-cell interactions. Consequently, many of the mechanisms discussed in this review are derived from studies performed in other malignancies, inflammatory diseases, or non-malignant experimental systems. While these observations may provide biologically plausible frameworks for understanding HMGB1 function in NPC, they should not be interpreted as evidence of experimentally validated NPC-specific pathways.

Throughout this review, we have explicitly distinguished among findings directly demonstrated in NPC, mechanisms established in EBV-associated or other biological systems, and hypothesis-generating concepts that require future validation. This distinction is particularly relevant for proposed links involving HMGB1-dependent regulation of macrophage polarization, C1q^+^ tumor-associated macrophages, exosomal HMGB1 signaling, TIM-3-associated pathways, tertiary lymphoid structure formation, and B-cell functional states. Future studies using NPC-specific experimental models and clinical specimens will be required to determine the extent to which these mechanisms operate in EBV-associated NPC. Accordingly, proposed HMGB1-associated mechanisms discussed in this review should be interpreted as a framework for future investigation rather than as established NPC signaling networks unless direct NPC-specific evidence is available.

### 7.2. The Dual Nature of HMGB1 in Physiology and Cancer

#### 7.2.1. Physiological Versus Pathological Functions

Although excessive HMGB1 signaling contributes to chronic inflammation, immune dysregulation, and tumor progression, HMGB1 also fulfills numerous essential physiological functions as discussed in Section 2. This functional duality represents one of the major challenges in developing HMGB1-targeted therapies. Extracellular HMGB1 also plays important roles in tissue regeneration and wound healing. Following tissue injury, HMGB1 released from damaged cells promotes recruitment of reparative cells and angiogenesis [122]. Consequently, broad HMGB1 inhibition could impair recovery of normal tissues after radiotherapy or surgery.

In antitumor immunity, HMGB1 exerts both stimulatory and suppressive functions depending on its redox state, receptor engagement, and cellular context. HMGB1 released during immunogenic cell death can facilitate dendritic cell maturation [123], thereby enhancing cytotoxic T cell priming. Interfering with these processes may unintentionally weaken antitumor immune surveillance. This phenomenon may be particularly relevant following radiotherapy and chemotherapy, where treatment-induced HMGB1 release can contribute to antigen cross-presentation and the generation of antitumor immune responses. HMGB1 additionally contributes to leukocyte trafficking and inflammatory cell recruitment [52]. While excessive leukocyte accumulation may support tumor-promoting inflammation, controlled immune cell recruitment is essential for effective host defense and antitumor immunity. Therefore, indiscriminate suppression of HMGB1 signaling may produce context-dependent immunosuppressive effects. 

#### 7.2.2. Context-Dependent Immune Effects

Beyond its dual physiological and pathological roles, another major unresolved challenge is understanding how HMGB1 generates diverse and, in some cases, opposing immune outcomes within the TME. The biological effects of HMGB1 depend on multiple factors, including its PTM profile, redox state, receptor engagement, and local metabolic context; however, the relative contribution of each factor remains incompletely understood. This complexity is exemplified by HMGB1-associated macrophage polarization, where studies have reported both M1-like and M2-like phenotypes. Whether these differences reflect distinct HMGB1 molecular states, microenvironmental influences, or limitations of current macrophage classification frameworks remains unclear. Future integration of PTM characterization with single-cell and spatial profiling approaches may help resolve these questions and support the development of more selective HMGB1-targeted therapeutic strategies.

#### 7.2.3. Spatial and Temporal Heterogeneity of HMGB1 Signaling 

Importantly, HMGB1 biology should be viewed within a spatial and temporal framework rather than as a uniform signaling process within the TME. Distinct HMGB1 molecular states may predominate in different anatomical niches, including hypoxic tumor cores, invasive margins, tertiary lymphoid structure-rich regions, necrotic areas, and the circulation. Likewise, the biological consequences of HMGB1 signaling are likely to evolve during disease progression and treatment, including baseline tumor growth, radiotherapy-induced tissue injury, post-treatment inflammation, tumor recurrence, and immunotherapy. Future spatially resolved and longitudinal studies will therefore be essential for defining how HMGB1 functions across different tumor compartments, treatment contexts, and clinical stages in NPC.

#### 7.2.4. Limitations of Current Clinical Evidence

An additional unresolved challenge relates to the clinical evidence supporting HMGB1 as a prognostic biomarker in NPC. Much of the available literature derives from relatively early observational studies, often involving limited patient cohorts and heterogeneous methodologies. Furthermore, the prognostic value of HMGB1 has not been extensively validated in contemporary NPC populations treated with current standards of care, including intensity-modulated radiotherapy and immunotherapy. As such, although elevated HMGB1 expression has been associated with adverse clinical outcomes, its utility as a clinically validated prognostic biomarker remains to be established through larger, well-annotated prospective studies.

### 7.3. Deciphering the Interplay of Concurrent PTMs

As reviewed in Section 3, individual PTMs, such as acetylation, phosphorylation, and oxidation, are well-documented to dictate whether HMGB1 functions as a nuclear chromatin-associated protein or an extracellular alarmin. However, a major limitation in current research is the tendency to evaluate these modifications in isolation. Within heterogeneous TME, HMGB1 is likely exposed to multiple concurrent signals, including hypoxia, lactate accumulation, oxidative stress, and viral oncoproteins. It remains completely unclear whether specific PTMs act competitively, sequentially, or synergistically on the same HMGB1 molecule. For instance, does site-specific N-glycosylation structurally mask or enhance downstream phosphorylation or other PTMs? Current liquid chromatography–tandem mass spectrometry (LC-MS/MS) techniques remain challenged by the detection of overlapping, multi-site modifications on endogenous proteins isolated from limited clinical specimens. Therefore, deciphering these combinatorial PTM patterns will be important for understanding how HMGB1 molecular states influence biological behavior in clinical settings. Notably, direct characterization of HMGB1 PTM states in NPC clinical specimens remains limited. Future development of PTM-resolved analytical approaches, including mass spectrometry-based and antibody-based methodologies, may help determine whether distinct HMGB1 modification patterns possess biomarker, patient-stratification, or therapeutic-monitoring value in NPC.

From a translational perspective, an important unresolved question is how to selectively neutralize the pathogenic extracellular activities of HMGB1 without disrupting its physiological functions within healthy tissues or its potentially beneficial roles in anti-tumor immunity. As discussed in Section 7.2, HMGB1 released during immunogenic cell death can contribute to dendritic-cell activation and T-cell priming, whereas chronic extracellular HMGB1 signaling may promote inflammation, immune suppression, and tumor progression. Consequently, future therapeutic strategies will require selective modulation of pathogenic HMGB1 signaling rather than indiscriminate suppression of all HMGB1 activity.

### 7.4. The Dilemma of Compartmentalized Therapeutic Selectivity

From a translational perspective, an important unresolved question is how to selectively neutralize the pathogenic extracellular activities of HMGB1 without disrupting its physiological functions within healthy tissues or its potentially beneficial roles in anti-tumor immunity. As discussed in Section 7.2, HMGB1 released during immunogenic cell death may contribute to dendritic-cell activation and anti-tumor T-cell priming, whereas persistent extracellular HMGB1 signaling has been associated with chronic inflammation, immune suppression, and tumor progression. Consequently, future therapeutic strategies will require selective modulation of pathogenic HMGB1 signaling rather than indiscriminate suppression of all HMGB1 activity. Nuclear HMGB1 contributes to the maintenance of genomic stability and participates in DNA repair pathways, including MMR and DSBR in normal cells. Systemic administration of direct neutralizers or global receptor antagonists may therefore carry risks of altering normal tissue homeostasis, particularly in highly regenerative tissues or in the lung, where RAGE expression is physiologically high [114]. Despite the potential benefits of GL, it is a pleiotropic small molecule with multiple off-target effects, including hypokalaemia and hypertension, which may contribute to systemic toxicities [124]. Furthermore, HMGB1-targeted antibodies may face tissue penetration barriers due to the dense and heterogeneous stromal architecture of NPC [125]. Developing tumor-targeted delivery systems, such as nanoparticles or antibodies that selectively recognize extracellular HMGB1 (e.g., disulfide or lactylated HMGB1) while sparing nuclear HMGB1 and preserving beneficial HMGB1-dependent immune functions, remains an important objective for future precision oncology.

An alternative and largely unexplored strategy may involve manipulating the extracellular redox state of HMGB1 to attenuate its biological activity. In principle, shifting HMGB1 toward its terminally oxidized (sulfonyl) form could reduce receptor engagement and downstream signaling, as terminally oxidized HMGB1 is generally considered biologically inactive. However, whether selective modulation of HMGB1 redox states can be achieved in vivo without affecting other redox-sensitive pathways, normal tissue function, or anti-tumor immune responses remains unclear and will require substantial further investigation. 

## 8. Future Perspective and Conclusions

An emerging therapeutic strategy lies in the spatial control of HMGB1, whereby PTM-dependent mechanisms are manipulated to selectively limit pathogenic extracellular HMGB1 signalling while preserving physiological and potentially beneficial immune functions. Modulating nucleocytoplasmic shuttling may reduce chronic HMGB1-driven inflammatory and immunosuppressive signalling while maintaining its normal chromatin-associated activities. For instance, EP has shown anti-tumor potential by restricting HMGB1 nucleocytoplasmic translocation through inhibition of the phosphorylation and hyperacetylation required for its nuclear release. This approach may reduce extracellular HMGB1 availability while preserving its physiological chromatin-associated functions.

Extracellular redox modulation of HMGB1 is another speculative avenue that warrants rigorous in vivo validation before clinical translation. In principle, shifting HMGB1 toward its terminally oxidized (sulfonyl) form could reduce receptor engagement and downstream signaling, as terminally oxidized HMGB1 is generally considered biologically inactive. However, whether selective modulation of HMGB1 redox states can be achieved in vivo without affecting other redox-sensitive pathways, normal tissue function, or anti-tumor immune responses remains unclear and will require substantial further investigation.

Beyond informing new HMGB1-directed therapies, PTM status may also influence the efficacy of existing HMGB1-targeting compounds. A notable example is GL, a direct HMGB1-binding inhibitor whose efficacy may be influenced by specific PTMs. For instance, molecular modeling studies have predicted that N-glycosylation of HMGB1 may reduce its binding affinity to GL, suggesting that compounds effective against unmodified HMGB1 in vitro may exhibit reduced efficacy in the highly modified and heterogeneous clinical TME [126]. However, this prediction remains to be experimentally validated using purified glycosylated HMGB1 and direct target-engagement assays. This observation highlights a potential challenge in drug development, namely that PTM-dependent structural variability may influence drug binding, target engagement, and therapeutic efficacy. 

Importantly, extracellular HMGB1 rarely functions as an isolated molecule in vivo. Instead, it can associate with chemokines, nucleic acids, nucleosomes, histones, extracellular vesicles, and other damage-associated molecules [36], thereby altering receptor engagement and downstream immune responses. This complexity may be particularly relevant in EBV-associated NPC, where viral and tumor-derived nucleic acids may co-operate with HMGB1 to influence innate immune sensing beyond the classical HMGB1–RAGE/TLR signaling. Furthermore, interpretation of mechanistic studies requires careful consideration of technical factors, including protein redox state, purification methods, aggregation status, and potential endotoxin contamination of recombinant HMGB1 preparations, all of which may substantially affect its reported biological activity.

Ultimately, successful development of HMGB1-targeted therapies will require a deeper understanding of the interplay between PTMs, structural conformation, and drug binding. Integrative multi-omics and structural profiling approaches will be important for understanding how EBV-associated stresses, hypoxia, and metabolic reprogramming dynamically reshape the HMGB1 PTM landscape. These context-dependent biochemical alterations present both opportunities and challenges for therapeutic development and may ultimately inform more precise treatment strategies for EBV-associated NPC.

## Figures and Tables

**Table 1:** Summary of HMGB1 post-translational modifications, modification sites, and reported biological effects.

PTM	Reported Biological Effects	Sites	Example of Reagent Induction	Ref.
Phosphorylation	Transportation toward cytoplasm by weakening DNA-binding affinity and disrupting structural folding stability	Ser35, 39, 42, 46, 53, and 181	Phorbol 12-myristate 13-acetate (PMA)	[29]
N-glycosylation	Enhances baseline nuclear mobility, nucleocytoplasmic exportation, and provides stability	Asn37, 134, and 135	(N/A)	[32]
O-GlcNAcylation	Reduction of HMGB1’s capacity to stabilize chromatin and repair DNA	Ser100, 107	(N/A)	[33]
Acetylation	Translocation into the cytoplasm and secretion	Lys2,6,7,11, NLS1 and NLS2	Lipopolysaccharide (LPS)	[34,35,47]
Oxidation	Extracellular shuttling	Cys23, 45, 106	Hydrogen peroxide (H_2_O_2_)	[36,37]
Methylation	Nucleocytoplasmic translocation	Lys42, 43, 112	(N/A)	[39,40,48]
ADP-ribosylation	Facilitates acetylation and works together for cytosolic translocation	Glu40, 47 and 179	LPS and alkylating agents that induce PARP1	[43,44]
Lactylation	Nuclear exportation	Lys177	Lactate	[45]
S-nitrosylation	Secretion	Cys106	LPS as inflammogen	[46]

Abbreviations: PARP1, poly(ADP-ribose) polymerase 1; N/A, not available or not reported.

**Table 2:** HMGB1-mediated immune cell interactions in cancer.

Cancer Type	Direct NPC Evidence	Immune Cell	Receptors	Outcome	Signaling	Evidence Type	Ref.
Osteosarcoma	No	Macrophage	RAGE	Promotes polarization to M2 macrophage	Not determined	Cell + Animal	[57]
Hepatocellular carcinoma (HCC)	No		TLR2	M2 macrophage polarization	TLR2-NOX2-autophagy axis	Cell + Animal	[58]
Oral squamous cell carcinomas	No		Not determined	M1 polarization	NF-κB/IL-6 signaling	Cell + Animal	[56]
Various cancer models	No	MDSCs	RAGE/TLR4	Promotes expansion, suppresses T cell activation	NF-κB, IL-10, ROS	Animal + Cell	[72]
Nasopharyngeal carcinoma (NPC)	Yes	CD8+ and CD4+ T cells	Not determined	Reduces T cell activity	NAT10-ac4C-DDX5-HMGB1	Patient + Cell + Animal	[75]
Head and neck cancer	No	T regulatory cell (Treg)	RAGE/TLR4	Attracts Treg migration and suppresses effector T cell proliferation	Not determined	Patient + Cell	[88]
Epithelial ovarian cancer	No	Exhausted T cells	TIM-3	T cell exhaustion	Modulate IL-32 expression through NFKB1 and TP53	Bioinformatics/Patient	[79]
Esophageal squamous cell carcinoma (ESCC)	No	Proliferating B cell	Not determined	Primes B cell migration to peritumor and induces angiogenesis	Proangiogenic marker expression, e.g., VEGF	Patient + Cell + Animal	[87]
HCC	No	B regulatory cell (Breg)	TLR2/4	Exosome-derived HMGB1 activates B cells and promotes Breg cell expansion	MAPK signaling pathway	Patient + Cell + Animal	[86]

**Table 3:** Preclinical Therapeutic Strategies Targeting the HMGB1 Axis in Nasopharyngeal Carcinoma.

Therapeutic Strategy/Agents	Primary Target/Proposed Mechanisms	Experiment Models	Key Outcomes	Ref.
Genetic silencing (shRNA/siRNA)	Downregulation of HMGB1 total transcript, disrupts lncRNA MIAT/HMGB1/IL6 axis	In vitro: C666-1, CNE-2 and HONE-1 cell line In vivo: nude mice xenograft	Suppresses cellular proliferation, overcomes cisplatin resistance and suppresses tumor growth	[101,102]
Glycyrrhizin (GL)	Physically binds HMGB1 A-box and B-box domains; blocks extracellular binding to RAGE/TLR4; disrupts HMGB1-Ku70 complexes	In vitro: HK-1 cell line	Sensitizes NPC cells to ionizing radiation and cisplatin	[90]

## Data Availability

No new data were created or analyzed in this study. Data sharing is not applicable to this article.
